# Feasibility of Mass Vaccination Campaign with Oral Cholera Vaccines in Response to an Outbreak in Guinea

**DOI:** 10.1371/journal.pmed.1001512

**Published:** 2013-09-10

**Authors:** Iza Ciglenecki, Keita Sakoba, Francisco J. Luquero, Melat Heile, Christian Itama, Martin Mengel, Rebecca F. Grais, Francois Verhoustraeten, Dominique Legros

**Affiliations:** 1 Médecins sans Frontières, Geneva, Switzerland; 2 Ministry of Health, Conakry, Guinea; 3 Epicentre, Paris, France; 4 Médecins sans Frontières, Conakry, Guinea; 5 World Health Organisation, Conakry, Guinea; 6 African Cholera Surveillance Network, Paris, France

## Abstract

Iza Ciglenecki and colleagues from Médecins sans Frontières report their experience of undertaking a mass vaccination campaign with oral cholera vaccines in response to an outbreak in Guinea.

*Please see later in the article for the Editors' Summary*

**Summary Points**

- Oral cholera vaccines are safe and effective, and in 2010 were added to WHO recommendations for cholera outbreak control. However, doubts about feasibility, timeliness, and acceptability by the population, and the fear of diverting resources from other preventive interventions, have discouraged their use during epidemics.
- We report on the first large-scale use of oral cholera vaccine as an outbreak control measure in Africa; this was also the first time Shanchol vaccine was used in Africa.
- We administered 312,650 doses of vaccine during two vaccination rounds in two coastal districts in Guinea. The feasibility, timeliness of implementation, and delivery cost were similar to those of other mass vaccination campaigns.
- The campaign was well accepted by the population, and high vaccination coverage was achieved despite the short time available for preparation, the two-dose schedule, the remote rural setting, and the highly mobile population.
- Oral cholera vaccines are a promising new tool in the arsenal of cholera control measures, alongside efforts to improve provision of safe water and sanitation and access to cholera treatment.

## Background

The number of reported cholera cases worldwide, as well as the frequency and scale of cholera epidemics, are increasing [1]. Traditional prevention measures, which focus on provision of safe water and proper sanitation, are undoubtedly the long-term solution for cholera control. But for populations in many low-income countries these measures remain out of reach: in Africa, 40% of families cannot access safe water and 60% have no access to appropriate sanitation [2]. Furthermore, once a cholera outbreak has started, these solutions are unlikely to be implemented fast enough or on a large enough scale to help control the spread. Nationwide epidemics, such as the recent one in Haiti—with over 600,000 cases and 7,000 deaths reported within the first 2 years [3]—highlight the urgent need for new tools and strategies.

Two oral cholera vaccines (OCVs) are currently licensed and prequalified by WHO: Dukoral (Crucell, Leiden, Netherlands), and Shanchol (ShanthaBiotechnics Ltd., Basheerbagh, Hyderabad, India). Both are given as a two-dose regimen and were shown to be safe and to provide sustained protection over several years [4]; Shanchol showed 66% efficacy over 3 years [5]. WHO recently updated its guidelines on cholera outbreak response to recommend considering OCV use in epidemic situations (as well as in endemic settings) [4].

However, ongoing questions and debate about the feasibility, cost, timeliness, and acceptability of reactive OCV campaigns have discouraged their use [6],[7]. Arguments against using OCV during epidemics have included: limited availability of vaccine; logistical challenges of rapidly transporting and delivering high volumes of cold-chain–requiring vaccines in resource-limited settings; difficulty achieving sufficient coverage with a two-dose regimen; acceptance of vaccination by the population; high vaccine cost; and fear of diverting limited resources from other control measures [6],[7]. Practical experience with OCV during epidemics has therefore remained limited to small-scale interventions in Asia [8]–[11].

Here we describe the implementation of the first large-scale reactive OCV campaign, conducted in Guinea between April and June 2012, and the first use of OCV Shanchol in Africa.

## Cholera Context in Guinea

Guinea, a country on the West African coast, regularly experiences cholera epidemics, with peaks occurring during the rainy season in July–August. The last major epidemic was in 2007, with 8,289 cases and 295 deaths [12]. However, in 2012 the first cholera cases were reported in February, long before the rainy season. As in previous epidemics, cases were first reported from the islands north and south of the capital, Conakry, in the Boffa and Forecariah districts. These islands are characterized by intense fishing activities and trade, a highly mobile population, limited access to health care, and poor access to safe water or basic sanitation.

The early start of the outbreak, together with a long inter-epidemic period and an ongoing cholera epidemic in neighboring Sierra Leone [13], suggested that a major epidemic was imminent. Considering these factors, the Ministry of Health of Guinea, with support of Médecins sans Frontières (MSF) decided in April 2012 to use OCV alongside already-implemented treatment and prevention strategies (health education; distribution of soap and chlorine for household water treatment).

## Implementation of the Vaccination Campaign

### 

#### Target population

The campaign focused on the coastal and island populations of the above-mentioned districts, which extend over about half the length of the Guinean coast: first, a population of 163,000 people in Boffa district, and 46,000 people in parts of Forecariah district (Kaback and Kakossa islands, and some neighboring ports on the mainland). Everyone older than 12 months presenting at vaccination sites was eligible to receive the vaccine during both vaccination rounds, which were spaced 2–3 weeks apart.

#### Vaccine procurement, storage, and transport

The bulk of the vaccine supply (320,000 doses) was shipped directly from the manufacturer in India, and 50,000 additional doses from MSF stock in Kampala, Uganda. The volume of the transport containers of vaccine was 29 m^3^. Vaccines were transported from Conakry's airport to the district capital in refrigerated trucks and stored in the field in refrigerated trucks or containers. Vaccines reached the field within 2 weeks of the order date.

Vaccine was supplied in individual vials, either in secondary packing of 35 vials or in individual secondary packing inside tertiary packing of 10 vials. One vaccine vial in the 35-vial package had a volume of 13.5 cm^3^, about five times greater than a dose of measles vaccine.

#### Vaccination teams

Forty-three teams composed of community members (community health workers, Guinean Red Cross volunteers, etc.) were assembled. Each team had a medical or paramedical leader and four to eight members, plus up to 12 helpers. Training for team leaders and members included a practice vaccination session.

#### Choosing vaccination sites

**Figure 1:**
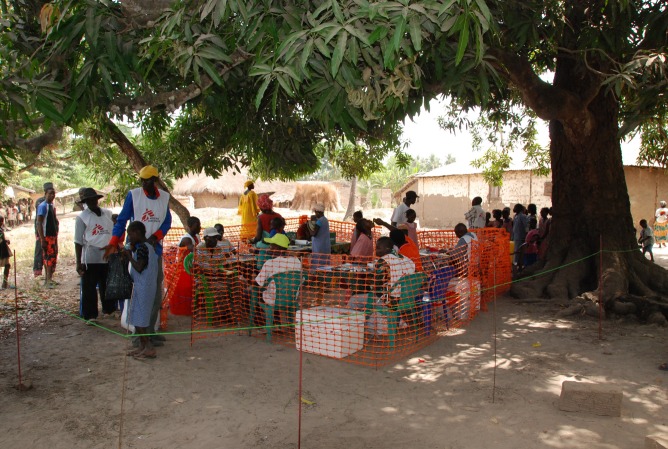
Vaccination team at work. *Image credit: David Di Lorenzo*.

Preliminary selection was done together with district medical authorities, then refined in consultation with community leaders. An important criterion was to keep travel distances short so that all family members, including elderly people and mothers with small children, could reach the sites easily. Altogether there were 287 sites, one per village or settlement (Figure 1).

#### Mobilizing the population

Due to the emergency nature of the intervention, the time period for social mobilization was short. The information was transmitted orally as described below; modern media were not used, as local radio or television are not available in the area and the mobile network coverage is low. Public awareness messaging included detailed information about the rationale of the campaign, the vaccine and the importance of two-dose schedule, along with standard cholera control messages regarding the necessity and availability of treatment and prevention measures. Existing material was used to illustrate the standard cholera control messages, but no special material was designed for the vaccination due to the limited amount of time available. Medical, administrative, and traditional authorities were informed in advance. Each community was visited 2 days before vaccination day by a health promoter, who provided educational and awareness information via village leaders. In more populated areas, local outreach workers conducted door-to-door mobilization.

#### Vaccination day

**Figure 2:**
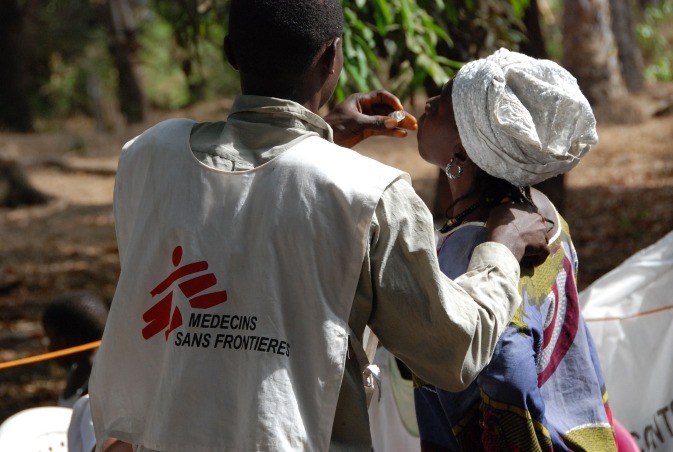
Administration of the vaccine. *Image credit: David Di Lorenzo*.

Each team had a car (two in Boffa) or boat to reach the vaccination sites. Vaccines were transported and used at ambient temperature on vaccination day. Vaccines leftover at the end of vaccination day were returned to the cold chain and used first on the following day. Before administration, the vaccine vial monitor (VVM) was checked for stability; the vial was shaken, opened, and administered or self-administered under observation (Figure 2). All VVM remained valid during the campaign.

To facilitate ingestion of the vaccine, we provided safe drinking water to each vaccinee (pre-packed 33 cl sachets from a Guinean manufacturer). Each vaccinee also received a vaccination card during the first round and was asked to bring the card for the second dose. However, during the second round we provided the vaccine to those who had lost their card or were not previously vaccinated.

In Forecariah, the second vaccination round was accompanied by distribution of preventive items (soap and chlorine solution for household water treatment), targeting women of childbearing age.

Teams vaccinated an average of 703 persons daily, up to 1,830 vaccinations/day/team. They spent several days in the larger villages but covered several smaller sites in one day. The vaccine wastage rate was below 1%. A total of 46 non-severe adverse effects were reported (mainly diarrhea and vomiting).

## Vaccination Coverage

Altogether 172,544 doses of vaccine were administered during the first round and 143,706 during the second. Based on administrative population figures, coverage with at least one dose (either first or second dose) was 92% in Boffa and 71% in Forecariah, and with the complete two-dose regimen was 68% in Boffa and 51% in Forecariah. However, a household survey conducted immediately after the campaign (Francisco Luquero, personal communication) found two-dose coverage in both areas to be about 76%, and one-dose coverage >90%. These differences are likely to be due to overestimation of actual population size by official figures.

### 

#### Time and costs

The complete campaign took 6 weeks from the decision to proceed until completion of the second round in Boffa (3-week interval between doses) and 5 weeks in Forecariah (2-week interval).

Cost per dose of vaccine delivered was US$2.89, including $1.85 for the vaccine itself and just over $1 for direct delivery costs (especially transport of teams and material, and payment for teams and other staff). Table 1 lists all costs that were factored into this calculation.

**Table 1:** Direct costs of mass vaccination campaign.

Item	Total (US$)	% Total
Vaccine ($1.85/dose)	585,063	64.0%
Water sachets ($0.036/sachet)	11,385	1.2%
Airfreight for vaccines	47,719	5.2%
Transit cost for vaccines	9,574	1.0%
Cold chain (truck rental, reparation of container in Boffa)	26,505	2.9%
Vaccination, supervision and sensitisation teams payments	63,308	6.9%
Training for the teams	4,899	0.5%
Small vaccination material and stationary, vaccination cards	13,705	1.5%
Logistic material, site preparation, waste management	13,333	1.5%
Transport cost (cars, trucks, boats and fuel)	139,851	15.3%
**Total**	915,341	100.0%
**Cost per dose delivered**	2.89	

Fixed administrative costs, MSF institutional costs, and costs linked to operational research are excluded.

#### Evolution of the epidemic

**Figure 3:**
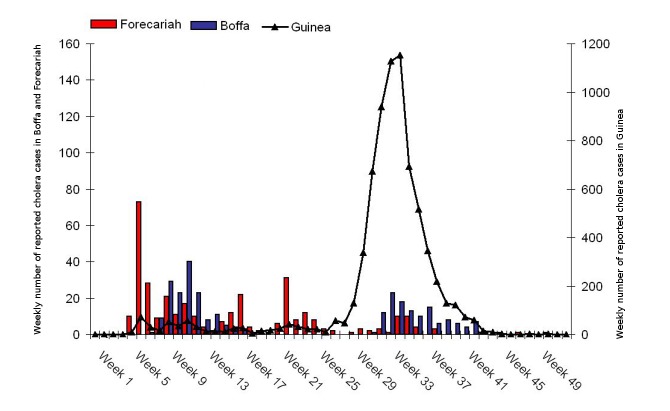
Weekly number of reported cholera cases in Guinea, and Boffa and Forecariah districts, Guinea, 2012. The vaccination campaign in Boffa took place on epidemiological weeks 13 and 16 and in Forecariah on weeks 22 and 24. *Source: Ministry of Health, Guinea*.

We were able to complete the vaccinations in two affected areas before the start of the seasonal cholera peak (Figure 3). The campaign's final outcomes will not be known until ongoing vaccine effectiveness and impact studies are completed; however, while the number of cholera cases peaked in other parts of Guinea during the rainy season, it remained at low levels in vaccinated districts (Ministry of Health, Cholera situation update, December 2012).

## Lessons for the Future

This experience demonstrated that mass campaigns with a two-dose OCV can be conducted successfully at the beginning of a cholera epidemic, even in a large, difficult-to-access area in Africa with a highly mobile population, and with little time for preparation of the campaign and social mobilization. Potential obstacles that discouraged earlier campaigns either failed to materialize or were quite manageable; in particular, the population was eager to get vaccinated during the outbreak, and logistical issues were resolved.

Ironically, in many ways our campaign was “over-resourced,” due to the anticipated obstacles. Vaccination teams in Boffa were over-sized (half-sized teams in Forecariah vaccinated the same number of people per day), which increased transportation needs. Transportation of water sachets was logistically challenging; although use of water is not necessary according to the manufacturer, we provided it to facilitate the intake of the salty-tasting vaccine. Vaccination cards were used only to verify vaccination status during the coverage survey. A simplified strategy without use of water and vaccination cards would reduce personnel and transport needs, and related costs.

Another potential simplification relates to vaccine vial presentation and packaging. The single-dose vaccines are voluminous, due partly to bulky secondary packaging. Additionally, the vaccine vial design is not ideal for oral use: single-dose vials are tiny, with metallic caps that are difficult to open.

There may also be potential to reduce cold chain needs. Although the vaccine is equipped with VVM 14 and considered temperature-stable, current labeling requires the vaccine to be stored in the cold chain. Documentation of thermostability is needed for future campaigns to be conducted using vaccines at ambient temperature.

A single-dose vaccine would also greatly simplify OCV campaigns. Studies in India found that partial immune response is achieved after a single dose [14], but whether this response is sufficient to confer clinical protection is not yet known. Similarly, a herd protection effect of Dukoral has been reported [15],[16], but its extent needs to be confirmed for Shanchol in additional settings.

Perhaps the most serious obstacles to wider use of reactive OCV campaigns are cost and limited supply of Shanchol. These constraints led us to drastically limit our target population to a small subset of those at risk; the full at-risk population includes everyone living along the coast of Guinea, including the capital (Conakry) with two million inhabitants, areas that were highly affected once the epidemic began. Funding for an OCV stockpile will be critical for the timely implementation of larger campaigns, an issue currently being addressed by WHO and its partners in an effort to improve OCV access for countries facing cholera outbreaks [17].

## Conclusion

Our experience demonstrates the feasibility of implementing OCV mass campaigns at the onset of major epidemics, similar to the campaigns with other vaccines used reactively (e.g., measles). OCVs are a promising additional tool for controlling cholera epidemics and should help prevent many illnesses and deaths, especially in settings with limited access to health care and where immediate improvements in sanitary conditions are improbable. In the near future, experience implementing OCV campaigns should be carefully documented, to provide future guidance for its most effective use.

## Supporting Information

Alternative Language Text S1 Article translated into French by Hélène Joguet.
(DOC)

## Abbreviations

OCV: oral cholera vaccine
MSF: Médecins sans Frontières
VVM: vaccine vial monitor
