# Supplementary material for: Feasibility of Mass Vaccination Campaign with Oral Cholera Vaccines in Response to an Outbreak in Guinea
Source: PLoS Med. 2013 Sep 10;10(9):e1001512. doi: 10.1371/journal.pmed.1001512 (PMC3769208; doi:10.1371/journal.pmed.1001512)
Supplement: Alternative Language Text S1 — Article translated into French by Hélène Joguet. (DOC) [file pmed.1001512.s001.doc]

**Faisabilité d’une campagne de vaccination de masse contre le choléra en réponse à une épidémie en Guinée**

Iza Ciglenecki^1^, Keita Sakoba^2^, Francisco J. Luquero^3^, Melat Heile^4^, Christian Itama^5^, Martin Mengel^6^, Rebecca F. Grais^3^, Francois Verhoustraeten^1^, Dominique Legros^1^

**Entités participantes :**

^1^Médecins sans Frontières, Genève, Suisse

^2^Ministère de la Santé, Conakry, Guinée

^3^Epicentre, Paris, France

^4^Médecins sans Frontières, Conakry, Guinée

^5^Organisation mondiale de la Santé, Conakry, Guinée

^6^Réseau Africain de Surveillance du Choléra, Paris, France

*Correspondance à adresser à : [Iza.Ciglenecki@geneva.msf.org](mailto:Iza.Ciglenecki@geneva.msf.org)

**Eléments de Résumé**

- Les vaccins anticholériques oraux sont sûrs et efficaces ; ils ont été ajoutés en 2010 aux recommandations de l’OMS en matière de lutte contre le choléra. Néanmoins, des doutes quant à la faisabilité, à l’opportunité et à l’acceptabilité par la population ajoutés à la crainte de détourner les ressources d’autres interventions préventives ont dissuadé de recourir à ce type de vaccins en périodes d’épidémies.
- Notre rapport présente la première utilisation à grande échelle du vaccin anticholérique oral en tant que mesure de lutte contre une épidémie de choléra en Afrique ; c’était également la première fois que le vaccin Shanchol® était utilisé en Afrique.
- Nous avons administré 312.650 doses de vaccin en deux tours de vaccination dans deux préfectures côtières de Guinée. La faisabilité, l’opportunité de la mise en œuvre et le coût de mise en œuvre ont été semblables à ceux d’autres campagnes de vaccination massives.
- La campagne a été bien acceptée par la population et une couverture vaccinale élevée a été atteinte en dépit du court délai de préparation, du schéma vaccinal prévoyant deux tours de vaccination, de la zone rurale reculée et de la forte mobilité de la population.
- Les vaccins anticholériques oraux apparaissent comme un nouvel outil prometteur dans l’arsenal des mesures de lutte contre le choléra, parallèlement aux efforts visant à améliorer la fourniture d’eau potable et d’assainissement et l’accès au traitement anticholérique.

**Contexte**

Le nombre des cas de choléra notifiés à l’échelle mondiale ainsi que la fréquence et l’ampleur des épidémies de choléra sont en progression [1]. Les mesures de prévention traditionnelles, principalement axées sur la fourniture d’eau potable et d’un assainissement adéquat, sont indubitablement, à terme, la solution pour lutter contre le choléra. Mais pour les populations de nombreux pays en développement, ces mesures restent hors de portée : en Afrique, 40% des foyers n’ont pas accès à l’eau potable et 60% n’ont pas accès à un assainissement adéquat [2]. De plus, une fois qu’une épidémie a débuté, ces solutions apparaissent peu susceptibles d’être mises en œuvre assez rapidement et large pour contribuer à maitriser l’épidémie. Les épidémies d’ampleur nationale, à l’image de celle qui a récemment frappé Haïti—avec plus de 600.000 cas et 7.000 décès notifiés sur les deux premières années [3]—montrent qu’il est urgent de se doter de nouveaux outils et stratégies.

Deux vaccins choléra oraux (VCO) sont actuellement brevetés et pré-qualifiés par l’OMS : le Dukoral® (Crucell, Leiden, Pays-Bas), et le Shanchol® (ShanthaBiotechnics Ltd., Basheerbagh, Hyderabad, Inde). Ces deux vaccins sont administrés selon un schéma en deux doses ; il a été démontré qu’ils sont sûrs et assurent une protection soutenue pendant plusieurs années [4]; le Shanchol® a démontré un taux d’efficacité de 66% sur trois ans [5]. L’OMS a récemment mis à jour ses directives relatives à la réponse aux épidémies de choléra et recommande désormais de recourir à la vaccination anticholérique orale en cas d’épidémie (ainsi que dans les contextes endémiques) [4].

Toutefois, les interrogations et le débat en cours à propos de la faisabilité, du coût, de l’opportunité et de l’acceptabilité des campagnes de vaccination anticholérique orale réactives ont dissuadé de les utiliser [6,7]. Les arguments avancés contre l’utilisation des VCO en période d’épidémie sont notamment les suivants : disponibilité limitée du vaccin ; défi logistique posé par la nécessité d’un acheminement rapide et par la livraison de forts volumes de vaccins requérant la chaine de froid dans des contextes faibles en ressources ; difficulté d’obtention d’une couverture suffisante dans le cadre d’un régime vaccinal à deux doses ; acceptation de la vaccination par la population ; coût élevé du vaccin, et crainte que les ressources limitées soient détournées d’autres mesures de lutte contre le choléra [6,7]. Dans un tel contexte, l’expérience de la vaccination anticholérique orale en cas d’épidémie est restée limitée à des interventions de faible ampleur en Asie [8-11].

Nous décrivons ici la mise en œuvre de la première campagne VCO de grande envergure menée en Guinée entre avril et juin 2012, et la première utilisation du VCO Shanchol® en Afrique.@

**Contexte du choléra en Guinée**

Pays situé sur la côte d’Afrique de l’Ouest, la Guinée est régulièrement confrontée à des épidémies de choléra, avec des pics qui surviennent pendant la saison des pluies en juillet – août. La dernière épidémie majeure s’est produite en 2007, avec 8.289 cas et 295 décès [12]. Cependant, en 2012, les premiers cas de choléra ont été signalés en février, bien avant la saison des pluies. Comme lors d’épidémies précédentes, les premiers cas ont été signalés dans les îles situées au nord et au sud de la capitale, Conakry, dans les préfectures de Boffa et de Forécariah. Ces îles sont caractérisées par des activités de pêche et de commerce intenses, par une population très mobile, par un accès limité aux soins de santé, et par un accès insuffisant à l’eau potable ou à l’assainissement de base.

Le fait que l’épidémie ait débuté tôt, ajouté à la durée importante de la période inter-épidémique et à la présence d’une épidémie de choléra en cours en Sierra Leone voisine [13] indiquait qu’une épidémie majeure était imminente. Tenant compte de ces différents éléments de contexte, le Ministère de la Santé de Guinée a décidé en avril 2012, avec le soutien de Médecins sans Frontières (MSF), de recourir à la vaccination anticholérique orale parallèlement au traitement et aux stratégies de prévention déjà en place (éducation sanitaire, distribution de savon et d’eau de javel pour le traitement de l’eau domestique).

**Mise en œuvre de la campagne de vaccination**

***Population cible :*** La campagne s’est concentrée sur les populations littorales et insulaires des préfectures mentionnées plus haut, lesquelles s’étendent sur presque la moitié de la bande littorale guinéenne avec, dans un premier temps, une population de 163.000 personnes dans la préfecture de Boffa, puis 46.000 personnes dans certaines parties de la préfecture de Forécariah (Iles de Kaback et de Kakossa, et certains ports voisins sur le continent). Toute personne âgée de plus de 12 mois se présentant à un site de vaccination était en droit d’obtenir une vaccination en deux tours, espacés de 2 à 3 semaines.

***Approvisionnement, stockage et transport des vaccins***

L’essentiel de l’approvisionnement en vaccin (320.000 doses) a été expédié directement depuis le site du fabriquant en Inde ; 50.000 doses supplémentaires ont été envoyées depuis le stock de MSF à Kampala, en Ouganda. Le volume des vaccins, y compris les emballages de transport, était de 29m^3^. Les vaccins ont été transportés depuis l’aéroport de Conakry vers la capitale de la préfecture dans des camions réfrigérés ; ils ont ensuite été stockés sur le terrain dans des camions ou des conteneurs réfrigérés. Dans les deux semaines suivant la date de commande, les vaccins étaient disponibles sur le terrain.

Le vaccin a été livré en doses individuelles, soit en conditionnements secondaires de 35 doses, soit en conditionnements secondaires individuels faisant partie d’un conditionnement tertiaire de 10 doses. Chaque dose de vaccin faisant partie du conditionnement secondaire de 35 unités a un volume de 13,5 cm^3^, soit environ 5 fois plus qu’une dose de vaccin contre la rougeole.

***Equipes de vaccination :*** 43 équipes de vaccination ont été constituées : elles comportaient des membres de la communauté (agents sanitaires communautaires, bénévoles de la Croix Rouge, etc.). Chaque équipe avait son propre responsable médical ou paramédical et comptait de 4 à 8 membres, et jusqu’à 12 aides. La formation des chefs d’équipe et des équipiers comportait notamment une séance pratique de vaccination.

***Choix des sites de vaccination:*** Un premier choix a été effectué en accord avec les autorités médicales de la préfecture avant d’être affiné en consultation avec les responsables de la communauté. Un critère important a été le maintien de faibles distances de déplacement afin que tous les membres de la famille, y compris les personnes âgées et les mères ayant des enfants en bas âge, puissent se rendre facilement sur le site de vaccination. Au total, le dispositif comptait 287 sites, un par village ou par campement (Figure 1).

***Mobilisation de la population* :** Compte tenu de l’urgence de l’intervention, on disposait de peu de temps pour la mobilisation sociale. Les messages de sensibilisation du public ont fourni des informations détaillées sur les raisons de cette campagne, sur le vaccin et sur l’importance du schéma en deux doses ; ils ont également diffusé des messages classiques sur la lutte contre le choléra, rappelant notamment que le traitement et les mesures de prévention étaient nécessaires et disponibles. Les autorités médicales, administratives et traditionnelles ont été informées au préalable. Chaque communauté a reçu, 2 jours avant la journée de vaccination, la visite d’un auxiliaire de santé venu informer et sensibiliser par l’intermédiaire des chefs de village. Dans les zones plus densément peuplées, les intervenants locaux chargés de la sensibilisation ont mobilisé la population en faisant du porte à porte.

***Journée de vaccination :*** Chaque équipe disposait d’une voiture (2 à Boffa) ou d’un bateau pour se rendre sur les sites de vaccination. Les vaccins ont été transportés et utilisés à température ambiante le jour de la vaccination. A la fin de la journée de vaccination, les vaccins non utilisés ont été replacés dans la chaine du froid et utilisés prioritairement le lendemain. Avant administration, la stabilité du vaccin a été vérifiée par pastille de contrôle de vaccin (PCV) ; chaque dose a été agitée, ouverte et administrée ou auto-administrée sous surveillance (Figure 2). Tous les tests de viabilité PCV sont restés valides pendant la campagne.

Afin de faciliter l’ingestion du vaccin, nous avons fourni de l’eau potable sûre à chaque personne vaccinée (sachets préemballés de 33cl fournis par un fabricant guinéen). D’autre part, chaque personne vaccinée s’est vu remettre une carte de vaccination pendant le premier tour de vaccination et il lui a été demandé d’apporter la carte pour la deuxième dose. Toutefois, lors du deuxième tour, nous avons fourni le vaccin y compris à ceux qui avaient perdu leur carte ou qui n’avaient pas fait l’objet d’une première vaccination.

A Forécariah, le second tour de vaccination a été accompagné d’une distribution d’articles de prévention (savon et solution chloré pour le traitement de l’eau domestique), ciblant les femmes en âge de procréer.

Les équipes ont vacciné en moyenne 703 personnes par jour, soit jusqu’à 1.830 vaccinations / jour / équipe. Elles ont passé plusieurs jours dans les villages les plus importants mais elles ont également couvert plusieurs petits sites dans une même journée. Le taux de gaspillage des vaccins a été inférieur à 1%. Au total, 46 effets indésirables sans gravité ont été notifiés (principalement diarrhée et vomissements).

### Couverture vaccinale

Au total, 172.544 doses de vaccin ont été administrées pendant le premier tour de vaccination et 143.706 pendant le second. Si l’on se fonde sur les données démographiques communiquées par l’administration, la couverture par au moins une dose (soit la première, soit la seconde dose) a été de 92% à Boffa et de 71% à Forécariah, et avec le schéma complet de deux doses de 68% à Boffa et de 51% à Forécariah. Pourtant, une enquête menée auprès des foyers immédiatement après la campagne (Francisco Luquero, communication) a montré que la couverture par deux doses dans les deux préfectures était de l’ordre de 76%, et la couverture par une dose >90%. Ces disparités sont probablement dues à une surestimation des données démographiques dans les chiffres officiels.

***Durée et coûts***: La campagne complète a duré 6 semaines -depuis la décision d’intervenir jusqu’à l’achèvement de la deuxième série de vaccination- à Boffa (intervalle de trois semaines entre les doses), et 5 semaines à Forécariah (intervalle de deux semaines).

Le coût par dose de vaccin délivré a été de USD 2,89, soit USD 1,85 pour le vaccin lui-même et un peu plus de USD 1 pour les coûts de livraison directe (en particulier le transport des équipes et du matériel et la rémunération des équipes et des autres membres du personnel). Le Tableau 1 présente la liste des coûts intégrés dans ce calcul.

***Evolution de l’épidémie.*** Nous avons pu effectuer les vaccinations dans deux zones touchées avant le début du pic saisonnier de choléra (Figure 3). Il faudra attendre l’achèvement des études d’impact et d’efficacité actuellement en cours pour connaître le bilan définitif de la campagne. Néanmoins, on peut d’ores et déjà constater qu’alors que le nombre de cas de choléra atteignait un pic dans d’autres parties de la Guinée pendant la saison des pluies, il est resté bas dans les préfectures vaccinées (Ministère de la Santé, Mise à jour situation du Choléra, décembre 2012).

**Enseignements pour l’avenir**

Cette expérience a démontré que des campagnes de vaccination de masse utilisant un vaccin anticholérique oral de deux doses peuvent être menées avec succès au début d’une épidémie de choléra, y compris dans une zone vaste et difficile d’accès à forte mobilité de population, et même lorsque l’on dispose de peu de temps pour préparer la campagne et sensibiliser la population. Quant aux obstacles potentiels qui avaient pu dissuader, dans le passé, de mettre en place des campagnes de ce type, soit ils ne se sont pas concrétisés, soit ils ont été plutôt gérables ; de fait, la population s’est montrée très désireuse de se faire vacciner pendant l’épidémie, et les questions logistiques ont été résolues.

Paradoxalement - notre campagne a bénéficié d’un excédent de ressources du fait des obstacles anticipés. Les équipes de vaccination de Boffa étaient surdimensionnées (des équipes deux fois moins importantes ont vacciné à Forécariah le même nombre de personnes par jour), ce qui a accru nos besoins de transport. D’autre part, le transport des sachets d’eau a représenté un défi logistique ; bien que l’utilisation de l’eau ne soit pas nécessaire selon le fabricant, nous en avons néanmoins fourni afin de faciliter l’ingestion du vaccin dont le goût est salé. Les cartes de vaccination ont été utilisées pour les besoins de l’enquête de couverture vaccinale, uniquement pour vérifier l’état de la vaccination. Une stratégie simplifiée sans utilisation d’eau et de cartes de vaccination réduirait les besoins de transport et de personnel, ainsi que les coûts correspondants.

La présentation et le conditionnement des doses de vaccin pourraient, de la même manière, faire l’objet d’une simplification. Les vaccins mono-doses sont volumineux, en raison notamment d’un conditionnement secondaire important. Par ailleurs, la présentation des doses de vaccin n’est pas vraiment adaptée à un usage oral : les vaccins mono-doses sont petits, et comportent un bouchon métallique difficile à retirer.

Il serait également possible de réduire les besoins en chaine de froid. Bien que le vaccin, équipé de PCV 14, soit considéré stable en température, l’étiquetage actuel exige un stockage en chaine du froid. Il conviendra de documenter la thermo-stabilité afin que les futures campagnes utilisent des vaccins à température ambiante.

D’autre part, un vaccin à administration en dose unique simplifierait considérablement les campagnes de vaccination orale. Or, des études menées en Inde ont montré qu’une réponse immunitaire partielle est obtenue après l’administration d’une seule dose, [14], mais on ne sait pas encore si cette réponse est suffisante pour conférer une protection clinique*.* De la même manière, un effet d’immunité de groupe du Dukoral® a été rapporté [15,16], mais sa portée reste à confirmer pour le Shanchol® dans d’autres contextes.

Les obstacles les plus sérieux à une généralisation des campagnes de vaccination anticholérique orale réactives sont peut-être les coûts et l’approvisionnement limité en Shanchol®. Ces contraintes nous ont conduit à restreindre notre population cible à une petite partie de la population à risque : en effet, la population à risque totale comprend toute personne vivant sur le littoral guinéen, y compris dans la capitale (Conakry) qui compte 2 millions d’habitants, dans les préfectures qui ont été fortement touchées une fois que l’épidémie avait débuté. Il sera impératif de financer l’achat d’un stock de vaccins oraux de manière à pouvoir lancer sans délai des campagnes de grande ampleur. Cette question est d’ailleurs actuellement étudiée par l’OMS et ses partenaires, soucieux d’améliorer l’accès à la vaccination orale dans les pays confrontés à des épidémies de choléra [17].

**Conclusion**

Notre expérience démontre la faisabilité de campagnes de masse de vaccination anticholérique orale au début d’épidémies majeures, à l’instar des campagnes qui sont menées avec d’autres vaccins utilisés à titre réactif (par exemple la rougeole). La vaccination anticholérique orale apparaît comme un outil supplémentaire prometteur dans la lutte contre les épidémies de choléra. Elle devrait contribuer à prévenir de nombreux cas et de nombreux décès, notamment dans des contextes caractérisés par un accès limité aux soins de santé et où une amélioration à court terme des conditions sanitaires est peu probable. A court terme, les expériences de mise en œuvre de campagnes de vaccination anticholérique orale devraient être soigneusement documentées, de manière à fournir des indications qui permettront d’y recourir de manière plus efficace.

**Remerciements**

Nous souhaitons remercier la population, les équipes de vaccination et les autorités de Boffa et de Forécariah pour leur enthousiasme et leur soutien dans l’organisation de la campagne. Nous remercions également Patricia Kahn pour son aide dans la préparation du manuscrit.

**Références**

1. World Health Organization (2011). Cholera, 2010. Wkly Epidemiologic Rec 31: 325-338. Disponible: <http://www.who.int/wer/2011/wer8631.pdf>. Dernier accès le 4 juillet 2013.
2. United Nations Children’s Fund and World Health Organization, 2012. Progress on drinking water and sanitation, 2012 update. Disponible: <http://www.unicef.org/media/files/JMPreport2012.pdf>. Dernier accès le 5 mars 2013.
3. Barzilay EJ, Schaad N, Magloire R, Mung KS, Boncy J, et al. (2013) Cholera surveillance during the Haiti epidemic – the first 2 years. N Engl J Med 368: 599-609. DOI: 10.1056/NEJMoa1204927
4. World Health Organization (2010). Cholera vaccines WHO position paper. Wkly Epidemiologic Rec 13: 117-128. Disponible: <http://www.who.int/wer/2010/wer8513.pdf>. Dernier accès le 5 mars 2013.
5. Sur D, Kanungo S, Sah B, Manna B, Ali M, et al. (2011) Efficacy of a low-cost, inactivated whole-cell oral cholera vaccine: results from 3 years of follow-up of a randomized, controlled trial. PLoS Negl Trop Dis 5(10): e1289. doi:10.1371/journal.pntd.0001289
6. Date K, Hyde T, Mintz E, Vicari A, Danovaro-Holliday MC, et al. (2011) Considerations for oral cholera vaccine use during outbreak after earthquake in Haiti, 2010-2011. Emerg Infect Dis 17: 2105-12. doi:[10.3201/eid1711.110822](http://dx.doi.org/10.3201%2Feid1711.110822)
7. Cumberland S (2009) An old enemy returns. Bull World Health Org 87: 85-6. Doi:10.2471/BLT.09.010209
8. Anh DD, Lopez AL, Thiem VD, Grahek SL, Duong TN, et al. (2011) Use of oral cholera vaccines in an outbreak in Vietnam: A case control study. PLoS Negl Trop Dis; 5: e1006. doi:10.1371/journal.pntd.0001006
9. [Calain P](http://www.ncbi.nlm.nih.gov/pubmed?term="Calain P"%5BAuthor%5D), [Chaine JP](http://www.ncbi.nlm.nih.gov/pubmed?term="Chaine JP"%5BAuthor%5D), [Johnson E](http://www.ncbi.nlm.nih.gov/pubmed?term="Johnson E"%5BAuthor%5D), Hawley ML, O’Leary M, et al. (2004) Can oral cholera vaccination play a role in controlling a cholera outbreak? Vaccine 22: 2444-51.
10. De Brettes A, de Carsalade GY, Petinelli F, Benoit Cattin T, Coulaud X, et al (2001). Le cholera à Mayotte. Bulletin Epidémiologique Hebdomadaire 8: 33-35.
11. Beatty ME, Jack T, Sivapalasingam S, Yao SS, Paul I, et al. (2004). An Outbreak of *Vibrio Cholerae* O1 infections on Ebeye Island, Republic of the Marshall Islands, associated with use of an adequately chlorinated water source. Clin Inf Dis: 38: 1-9.
12. World Health Organisation (2013) Cholera country profile: Guinea. Available: <http://www.who.int/cholera/countries/CountryProfileGuinea2009.pdf> Dernier accès le 5 mars 2013.
13. World Health Organization (2012) Outbreak bulletin 2:2. Available: <http://www.afro.who.int/en/clusters-a-programmes/dpc/epidemic-a-pandemic-alert-and-response/outbreak-news/3690-cholera-in-sierra-leone-update-18-september-2012.html>. Dernier accès le 5 mars 2013.
14. Kanungo S, Paisley A, Lopez AL, Bhattacharya M, Manna B, et al. (2009) Immune responses following one and two doses of the reformulated, bivalent, killed, whole-cell, oral cholera vaccine among adults and children in Kolkata, India: a randomized, placebo-controlled trial. Vaccine 27: 6887-93.
15. Ali M, Emch M, von Seidlein L, Yunus M, Sack DA, et al. (2005) Herd immunity conferred by killed oral cholera vaccines in Bangladesh: a reanalysis. Lancet 366: 44–49. doi:10.1016/S0140-6736(05)66550-6[

](http://www.thelancet.com/popup?fileName=cite-using-doi)
16. Khatib AM, Ali M, Seidlein L, Kim DR, Hashim R, et al. (2012) Effectiveness of an oral cholera vaccine in Zanzibar: findings from a mass vaccination campaign and observational cohort study. Lancet Infect Dis 12: 837-844. doi:10.1016/S1473-3099(12)70196-2[

](http://www.thelancet.com/popup?fileName=cite-using-doi)
17. Martin S, Costa A, Perea W (2012) Stockpiling oral cholera vaccine. Bull World Health Organ 90: 714. doi: 10.2471/BLT.12.112433

**Figure 1. Equipe de vaccination au travail. Crédit photo : David Di Lorenzo**

**Figure 2. Administration du vaccin. Crédit photo : David Di Lorenzo**

**Figure 3. Nombre hebdomadaire de cas de choléra notifiés en Guinée, et dans les préfectures de Boffa et de Forécariah, Guinée, 2012. La campagne de vaccination de Boffa a eu lieu dans les semaines 13 et 16 et celle de Forécariah dans les semaines 22 et 24. Source: Ministère de la Santé, Guinée.**

**Tableau 1. Coûts directs de la campagne de vaccination massive***

|  | **Total (en US$)** | **% total** |
| --- | --- | --- |
| Vaccins ($1,85 / dose) | 585.063 | 64,0% |
| Sachets d’eau ($0,036 / sachet) | 11.385 | 1,2% |
| Transport aérien vaccins | 47.719 | 5,2% |
| Coûts de transit vaccins | 9.574 | 1,0% |
| Chaine de froid (location de camions, réparation de conteneur à Boffa) | 26.505 | 2,9% |
| Rémunération des équipes de sensibilisation, de supervision et de vaccination | 63.308 | 6,9% |
| Formation des équipes | 4.899 | 0,5% |
| Papeterie et petit matériel de vaccination, cartes de vaccination | 13.705 | 1,5% |
| Matériel logistique, préparation des sites, gestion des déchets | 13.333 | 1,5% |
| Coûts de transport (voitures, camions, bateaux et carburant) | 139.851 | 15,3% |
| **Total** | 915.341 | 100,0% |
| **Coût par dose délivrée** | 2,89 |  |

** Les coûts fixes administratifs, les coûts institutionnels MSF et les coûts liés à la recherche opérationnelle sont exclus.*
